# Clazakizumab: A Therapeutic Approach for Patients With Late Antibody-Mediated Rejection

**DOI:** 10.1016/j.ekir.2025.09.040

**Published:** 2025-10-08

**Authors:** Stanley C. Jordan

**Affiliations:** 1 Department of Medicine, Comprehensive Transplant Center, Cedars-Sinai Medical Center, Los Angeles, California, USA

See Translational Research on Page 4027

Modification of the interleukin-6(IL-6)/IL-6 receptor signaling has emerged as an important therapeutic target for the treatment of autoimmune and inflammatory diseases, including multiple nonrandomized reports of efficacy in the treatment of antibody-mediated rejection (AMR) in kidney transplant recipients.^1^, ^2^, ^3^, ^4^, ^5^ However, the mechanism(s) of action associated with the perceived benefit have not been clearly elucidated. The article by Zhang *et al.*^6^ appearing in this issue of *KI Reports* presents a novel assessment of peripheral blood and renal biopsy tissue obtained from patients participating in a randomized, placebo controlled clinical trial of clazakizumab (CLZ) (an anti–IL-6 antibody) for the treatment of late-stage AMR in kidney transplant recipients.^7^ Here, the authors used peripheral blood transcriptomics to assess gene expression in patients with AMR receiving placebo versus CLZ. Importantly, they identified a decrease in IL-6–associated *JAK-STAT* pathway genes signaling with CLZ, and a reduction in genes that enriched for T follicular helper cell (Tfh) and activated platelet signatures. These are well-known pathways for IL-6 activation of immune and inflammatory events as well as donor-specific human leucocyte antigen antibody (DSA) generation, which showed inhibition by CLZ. Some patients appeared to lose the benefit of CLZ therapy over time; however, this did not correlate with loss of efficacy in stabilizing estimated glomerular filtration rate (eGFR). Our open label study using CLZ to treat AMR confirmed stabilization of eGFR as well.^8^ The authors found 1 peripheral gene module that correlated with the MMDx AMR score that indicated enriched monocyte signature genes and Fcg receptor–mediated effector functions as well as leukocyte transendothelial migration. This is an important finding because it suggests that monitoring patients’ blood for these genes could possibly be used to diagnose and follow modification of immune activation events by CLZ and other immune modulatory agents. In addition, the authors found that CLZ treatment was associated with significant reductions in damaged renal tubule gene signatures and preservation of podocyte signatures.

These findings are novel and of great interest to the transplant community and the authors are to be congratulated for their efforts. Importantly, the peripheral blood gene signatures were integrated with the kidney microarray data generated as part of the original trial MMDx biopsy end points.^7^ Their aim was to determine the effects of IL-6 blockade on circulating immune cells, and the extent to which these reflected molecular changes in the allograft, and whether this information would provide insights into why CLZ treatment might or might not show efficacy in the treatment of AMR. We know that DSAs promote allograft inflammation and tissue injury through direct binding to endothelial cells. However, injury is mediated through complement-dependent cellular cytotoxicity and antibody-dependent cellular cytotoxicity.^9^ Here, antibody-dependent cellular cytotoxicity is mediated by Fc-mediated effector pathways dependent on FcgR-expressing immune cells, such as monocytes, macrophages, and natural killer cells within the graft. Importantly, the authors showed that CLZ treatment reduced the expression of immune pathways related to Fc-mediated effector functions of antibody, including Fcg receptor–mediated phagocytosis and natural killer cell–mediated cytotoxicity within peripheral blood. This suggests that immune cells activated within the graft vasculature can be detected in the peripheral blood via transcriptomics. As previously mentioned, the authors demonstrate that CLZ treatment was associated with significant downregulation of peripheral blood gene sets, including B cell activation, JAK-STAT pathway genes, and Tfh signatures. Here, Tfh cells are critical for the progression of germinal center responses and the generation of high-affinity DSAs, responsible for the clinical features of AMR.

IL-6 is required for the differentiation of CD4 T cells into polarized Tfh. The authors conclude that effects of CLZ on Tfh may contribute to the reduction in DSA observed in the phase 2 study. In support of these observations, our group performed a desensitization study with CLZ, which showed reductions in human leucocyte antigen antibodies in 20 sensitized patients allowing all to be transplanted.S1 Importantly, after transplant, CLZ was continued monthly. Here, we saw no *de novo* DSAs and only 1 patient had a demonstrable DSA at 12 months posttransplant. Immunity monitoring showed progressive declines in Tfh cells and dramatic increases in regulatory T cells and regulatory B-cells at 6 to 12 months posttransplant.

IL-6 is critical for Tfh development and sustenance. A recent report clarified this process. Papillion *et al.*S2 showed that IL-6 produced by antigen-presenting cells stimulates Tfh activation with subsequent B-cell activation. In molecular analysis of these events, the authors demonstrated that IL-6 did not inhibit IL-2 production but inhibited JAK/STAT 5 activation responsible for expression of IL-2Rb, thus preventing the utility of IL-2 in Tfh cells and permitting the progressive inflammatory and immune activation events to proceed. In this regard, CLZ treatment should inhibit Tfh responses by inhibiting the effect of IL-6 on Tfh development and allow IL-2/IL-2R utility to deviate these cells to regulatory T-cells.

The authors also examined renal tissue obtained from patients in this study. Here, they found genes associated with damaged tubular and progenitor signatures, possibly indicating renal tubular cell injury and attempts at repair following injury were downregulated by long-term CLZ. In addition, a podocyte gene–rich module was preserved with CLZ but downregulated with placebo, indicating a process of preservation of glomeruli with CLZ. These findings are of great interest because they suggest that inhibition of IL-6–dependent immune activation can result in improved renal functional capacity, which was seen in the phase 2 study. However, there is likely an alternative explanation. Fogal *et al.*,S3 using a humanized mouse model of human coronary artery rejection demonstrated significant increases in IL-6 gene expression in the endothelial cells of the coronary arteries. Adoptive transfer of human peripheral blood mononuclear cells allogeneic to the artery results in T-cell infiltrates and intimal expansion 4 weeks later. Anti–IL-6 (human) significantly reduced the magnitude of intimal expansion and total T-cell infiltration, as well as Th17 markers while increasing regulatory T-cell infiltration. Lion *et al.*S4 have also examined the role of IL-6 in endothelial cell immunogenicity. Blockade of IL-6 reduced endothelial cell secretion of IL-6. Preactivation of endothelial cells by anti–human leucocyte antigen or DSA binding increased IL-6 secretion, that was further increased by concurrent binding of both antibodies. The investigators showed that this was inhibited by anti-IL-6. Thus, it is possible that CLZ-treated patients might have benefited by inhibition of IL-6 secretion in endothelial cells of the allografts that resulted in increased gene signals for podocyte healing.

There are many thoughts that I have regarding this study. First, how would these monitoring techniques be used in a clinical trial and how would they relate to a clinical end point; specifically eGFR pre- or post-CLZ treatment. One of the first studies in AMR treatment was carried out after phase 2 data from initial studies indicated a benefit in stabilizing eGFR with CLZ.^5^^,^^7^ Unfortunately, the phase 3 study (IMAGINE) failed to show any difference in eGFR decline between placebo versus CLZ-treated patients. Although results have not been published, a possible explanation for failure is the use of a 50% reduced dose of CLZ (12.5 mg vs. 25 mg) monthly for the phase 3 study. Test monitoring efficacy of IL-6 inhibition rely on reductions of C-reactive protein. However, this may not be a reliable indicator of inhibition of the effector function of IL-6 inhibition on immune cells and endothelium. Here, the data presented by Zhang *et al.*^6^ have potential significance in monitoring therapeutic approaches to treatment of AMR.

## Disclosure

SCJ reports grants and intellectual property rights from CSL-Behring, grants from Hansa Biopharma; and grants and consultation fees from Argenx.

## Supplementary Material

Supplementary File (PDF) **Supplementary****References.**

## Footnotes

Supplementary File (PDF)
Supplementary References.
