# Supplementary material for: Clazakizumab: A Therapeutic Approach for Patients With Late Antibody-Mediated Rejection
Source: Kidney Int Rep. 2025 Oct 8;10(11):3741–3. doi: 10.1016/j.ekir.2025.09.040 (PMC12640071; doi:10.1016/j.ekir.2025.09.040)
Supplement: Supplementary File (PDF) — SupplementaryReferences. [file mmc1.pdf]

S1. Vo AA, Huang E, Ammerman N, Toyoda M, Ge S, Haas M, Zhang X, Peng A, Najjar R, Williamson S, Myers C, Sethi S, Lim K, Choi J, Gillespie M, Tang J, Jordan SC. Clazakizumab for desensitization in highly sensitized patients awaiting transplantation. Am J Transplant. 2022 Apr;22(4):1133-1144. doi: 10.1111/ajt.16926..

S2. Papillion A, Powell MD, Chisolm DA, Bachus H, Fuller MJ, Weinmann AS, Villarino A, O'Shea JJ, León B, Oestreich KJ, Ballesteros-Tato A. Inhibition of IL-2 responsiveness by IL-6 is required for the generation of GC-T_{FH} cells. Sci Immunol. 2019 Sep 13;4(39):eaaw7636. doi: 10.1126/sciimmunol.aaw7636.

S3. Fogal B, Yi T, Wang C, Rao DA, Lebastchi A, Kulkarni S, Tellides G, Pober JS. Neutralizing IL-6 reduces human arterial allograft rejection by allowing emergence of CD161+ CD4+ regulatory T cells. J Immunol. 2011 Dec 15;187(12):6268-80. doi: 10.4049/jimmunol.1003774.

S4.Lion J, Maitre ML, de Truchis C, Taupin JL, Poussin K, Haziot A, Chong E, Glotz D, Mooney N. Restriction of interleukin-6 alters endothelial cell immunogenicity in an allogenic environment. Clin Transplant. 2023 Mar;37(3):e14851. doi: 10.1111/ctr.14851.
